# The role of proprioception in the management and rehabilitation of adolescent idiopathic scoliosis

**DOI:** 10.3389/fresc.2026.1806503

**Published:** 2026-04-02

**Authors:** Ting Zhang, Qian Zhou, Lulu Shen, Song Gao

**Affiliations:** 1 College of Physical Education and Health Sciences, Zhejiang Normal University, Jinhua, China; 2 University Hospital, Zhejiang Normal University, Jinhua, China; 3 Physical Education Teaching Group, The First Experimental School of Shijiazhuang High-tech Industrial Development Zone, Shijiazhuang, China; 4 Jinhua Deren Rehabilitation Equipment Corporation, Jinhua, China

**Keywords:** adolescent idiopathic scoliosis, idiopathic scoliosis, proprioception, rehabilitation, scoliosis

## Introduction

Scoliosis is defined as a three-dimensional abnormal torsion of the spine and trunk (1). Adolescent scoliosis represents the most prevalent form of scoliosis, affecting approximately 3% of school-aged children globally. The aetiology of adolescent scoliosis remains unclear, and patients typically present without skeletal abnormalities or neuromuscular diseases (2). Consequently, adolescent scoliosis is also referred to as AIS (3). AIS is a multifactorial disease, with a potential aetiology encompassing heredity, living environment, biomechanics, neurology and growth (4). Nevertheless, it remains unclear whether these factors are the primary cause of AIS or merely secondary abnormal manifestations. AIS exhibits a gender tendency, with a male-to-female ratio of approximately 1:8 (5). Adolescents undergo rapid growth and development, which results in a notable worsening of the scoliosis. Additionally, they may experience back pain and cardiopulmonary dysfunction. AIS can be treated via surgical or conservative means. The conservative treatment options include brace fixation, joint loosening, massage, electrical stimulation and functional training (6). The efficacy of braces as a therapeutic intervention has been substantiated; however, the potential adverse effects are becoming increasingly evident (7). Currently, the treatment of AIS is primarily focused on functional training, with particular emphasis on proprioceptive training, which has emerged as a prominent area of research.

Proprioception can be defined as the sensory information transmitted to the central nervous system by muscles, tendons and joints as a result of changes in direction, spatial position and speed (8). In the event of spatial alterations in the motor organs, the proprioceptors therein are stimulated to transmit signals to the central nervous system (9). The proprioceptive system is instrumental in guiding the brain's adjustments to posture and its decisions regarding motion (10). However, it is possible that abnormal proprioceptive input may act on the brain independently, that is to say that abnormal sensory input may result in the triggering of abnormal muscle tension. The proprioceptive system plays a pivotal role in maintaining the normal physiological curvature of the spine, spinal stability, and the ability to support weight and maintain posture (11). This may be attributed to an imbalance between mechanical load and the proprioceptive system, which is a potential consequence of adolescent scoliosis. Patients with scoliosis exhibit more pronounced physical balance dysfunction and require a greater expenditure of time to stabilise their posture than individuals without the condition (12). Proprioceptive training has been demonstrated to enhance balance ability and improve muscle feeling and ability.

An anomalous proprioceptive system is intimately associated with AIS, and its underlying pathological mechanism remains a topic of active investigation (13). A more precise definition of the proprioceptive system in AIS is of great significance for the establishment of a more scientific diagnostic and treatment system. This article provides a concise overview of the characteristics of the proprioceptive system in AIS, emphasising the pivotal role that proprioception plays in its development. The objective of this study is to extend the existing rehabilitation approach for AIS by incorporating proprioceptive training into clinical diagnoses, treatments and rehabilitation strategies.

## Abnormal proprioception system may promote AIS

Abnormal proprioception may be the sole distinguishing factor between AIS and other forms of scoliosis (14). A study identified notable discrepancies between the AIS and control groups with regard to position and vibration perception as measured by somatosensory evoked potentials. However, these characteristics were not observed in patients with congenital scoliosis (15). However, the study did not employ a pre- and post-control model to induce scoliosis, which precludes an explanation of whether the abnormality of the proprioception system is the cause of AIS. It is therefore necessary to conduct further research in order to determine whether proprioception is the underlying cause or a secondary manifestation of AIS.

Abnormal proprioception may be a potential contributing factor in the aetiology of AIS. In order to induce scoliosis in mice, researchers eliminated proprioceptor-related genes (11, 16). In a particular study, the regulator Runx3 or Egr3 of the proprioception system was genetically modified, resulting in the establishment of a mouse model manifesting characteristics analogous to those observed in scoliosis was successfully established (11). Another study utilised gene editing to target Piezo2, a mechanically sensitive ion channel protein, which resulted in the induction of spinal misalignment and hip dysplasia (16). The present findings suggest that mutations in Runx3, Egr3 and Piezo2 may be a potential cause of AIS (16, 17). Consequently, it can be hypothesised that abnormal proprioception is significantly associated with the development of AIS.

The pathways of the afferent nerves involved in the control of posture are distinct (18). Simoneau et al. (19) observed that AIS relies more heavily on ankle proprioception for the control of static balance, with the resulting imbalance becoming more pronounced in dynamic conditions. This finding is consistent with the research of Leberre et al. (20), who observed that the disorder of the dynamic proprioception system in AIS is more pronounced. The proprioceptive pathway of AIS differs from that of healthy individuals, with potential issues in the input and central integration processes.

The proprioceptive abnormality of AIS is not only reflected in receptors and conduction pathways, but also manifests as a distinct central integration ability compared to that observed in individuals without the condition (19). As posited by the theory of sensory reweighting, when sensory information is disrupted, the central nervous system adjusts the relative importance of different sensory inputs to help the body adapt to changes in sensory information. Current findings suggest that the sensory reweighting mechanism is impaired in patients with AIS. In a study examining ankle proprioceptive reintegration in AIS patients, the subjects experienced more difficulty than the control group in reweighting ankle proprioceptive information following brief sensory deprivation (21). In this study, ankle proprioceptive reweighting resulted in increased central pressure rate variability in AIS patients with severe spinal deformity, regardless of visual availability. Several studies have assessed the ability of AIS patients to process visual information in order to control body sway. When both the visual and somatosensory systems are challenged simultaneously, AIS patients exhibit greater body sway than control groups (21–23). It is likely that the central nervous system relies more heavily on vestibular input to regulate balance under these circumstances. The greater body sway may result from changes in vestibular reweighting triggered by higher variability in vestibular information.

Haumont et al. (23) observed that the central sensory adjustment system of AIS patients is less efficient. Individuals diagnosed with AIS have been shown to exhibit a slower response time in initiating foot tendon vibration compared to healthy controls. However, they have also been demonstrated to exhibit a higher frequency in initiating and executing hip strategies. The increased incidence of head shaking observed in AIS patients during dynamic balance tasks indicates a heightened involvement of the vestibular system in posture control (24). This finding is in consistent with the results reported by Pialasse et al. (25). In the event of an imbalance in sensory integration in AIS, the sequence of local muscle activation is inverted (26). Nevertheless, the phenomenon of central integration compensation resulting from a proprioceptive abnormality appears to manifest primarily in adolescents, rather than in adults. In their study, Lambert et al. (27) conducted unilateral labyrinthine resection on tadpoles and adult grass frogs. It was observed that the tadpoles exhibited pronounced scoliosis, whereas the posture of the adult grass frogs returned to normal and the bones showed no significant deformities. This may be associated with the complete maturation of the proprioception system and the development of the nervous system in the frog following its transition from an aquatic to a terrestrial habitat. However, the formation of AIS is influenced by a multitude of internal and external environmental factors. Those that impede the normal development of the spine may exacerbate spinal imbalance. It is therefore possible that AIS may also manifest in adults.

## Abnormal proprioceptive system will be accompanied by AIS deterioration

An imbalance of the sensory system is frequently regarded as a potential cause of poor posture, preceding an analysis of the underlying biomechanical structure (28). However, the persistence of spinal posture abnormalities does not necessarily indicate stability in morphology and function. In the process of spinal adaptive compensation, an imbalance in the proprioception system is evident, particularly in the paraspinal muscles of the spine (29).

The muscle function of individuals with AIS is closely associated with the paraspinal muscles. In addition to reflecting the biomechanical function of an effector in the reflex arc of human posture, paraspinal muscles also feed back local information to the centre for sensory reorganization (30). Consequently, the function of the paraspinal muscles provides an intuitive embodiment of the running ability of the proprioceptive system. The sustained poor posture associated with AIS has resulted in the formation of stable yet erroneous neuroplasticity (31). The continuous feedback of aberrant proprioceptive signals from the AIS spinal muscles to the central nervous system (CNS) results in a loss of the CNS's capacity to accurately regulate peripheral sensation. The erroneous neural plasticity may prompt the central nervous system to allocate an inappropriate sensory weight distribution, thereby generating an erroneous posture control mode (32). The proprioceptive input cannot be promptly adjusted, and the posture of AIS will be challenging to recuperate. This imbalance of the multisensory system, which is predominantly influenced by proprioception, will be further exacerbated.

The proprioception of AIS paraspinal muscles differs from that of the normal spine. The imbalance of AIS paraspinal muscles is reflected in the shape, type and EMG signal of muscle fibres, and its characteristics are outlined in Table 1 (33). Furthermore, PAX3, a susceptible gene that regulates the development of paraspinal muscles; TGFB1, a modifier gene that regulates spinal curvature; and LBX1, a transcription factor that is crucial to muscle quality, have all been demonstrated to be significantly correlated with AIS (34, 35). A reduction in the expression of the transcription factor SOX9 during chondrocyte differentiation can result in the development of scoliosis and progressive disc degeneration, which is associated with advancing age (36). The discrepancy in the tissue composition of AIS paraspinal muscles is exacerbated with the progression of lateral curvature. This indicates a further deterioration in the proprioceptive system, which is accompanied by an abnormal function of the AIS paraspinal muscles.

**Table 1:** Characteristics of paraspinal muscle imbalance in AIS.

Characteristics	Description
(1) The distribution of paraspinal muscle fiber types on the concave-convex side of AIS.	The type I muscle fibers of paraspinal muscles increased obviously, and the type II muscle fibers shrank relatively, which was more obvious on the convex side (37);
(2) The relationship between the scoliosis and the types of paraspinal muscle fibers.	The severity of AIS may be positively correlated with the content of type I muscle fibers in the convex paraspinal muscles (38);
(3) The difference of EMG signals between concave and convex sides of AIS paraspinal muscles.	The recruitment degree of EMG signals in the convex side of spine is obviously higher than that in the concave side, and the root mean square value of paravertebral muscles in the convex side is higher than that in the concave side during isometric contraction (39);
(4) Morphological differences of paraspinal muscles on both sides of AIS.	Collagen content in concave muscle is more (40), and its muscle tension and stiffness are obviously greater than those in convex muscle (33), which is closely related to Cobb Angle (41);
(5) The mechanical stiffness of AIS intervertebral disc is decreased and the annulus fibrosis occurs.	It is secondary to the change of mechanical factors after scoliosis, but not the primary cause (42, 43).

## Proprioceptive relearning will help to improve AIS

In the event of an erroneous proprioceptive input, it is not possible to make an adjustment, which in turn makes it challenging to recover the correct posture and movement for an AIS individual. In accordance with the law of motor function reacquisition, the accurate input of proprioception is a fundamental prerequisite for the anticipated movement (26). It has been demonstrated that enhancing the body's proprioceptive ability represents the initial stage in ensuring the control of trunk movement and posture in individuals with scoliosis (44, 45). Modifying the trigger area, pressure direction, and joint angle can enhance the input of proprioception, thereby improving posture control. It is therefore of great significance to correct the proprioception of individuals with AIS (46).

The comprehensive training programme for AIS primarily entails modifications to joint position and muscle function, with the objective of facilitating relearning of the proprioceptive system. This encompasses a range of techniques, including Schroth exercise, functional individual therapy, and lateral movement (47). Other forms of treatment, such as the use of assistive devices (including bracing and orthopaedic insoles), acupuncture and massage, functional electrical stimulation and core training, may also be employed (46). These methods are closely associated with alterations in local ontological sensory information. A brace is a biomechanical device that is used to correct spinal posture, and it is a common treatment for AIS. The use of braces can facilitate the adaptation of local tissues to the altered mechanical environment, thereby providing continuous feedback of new proprioceptive information to the central nervous system and facilitating the correction of scoliosis (44).

In a study by Monticone et al. (48), a task-oriented training programme was devised which included proprioception training. A total of 110 children with mild scoliosis were randomly assigned to either a proprioceptive training group (experimental group, 55 cases) or a traditional spinal exercise group (control group, 55 cases). The findings indicated that the proprioceptive training group exhibited a notable enhancement in Cobb angle, thereby demonstrating superior outcomes in comparison to the traditional training regimen. The beneficial effects of this intervention have been observed to persist for at least one year following its cessation. Another study (49) demonstrated that students who received holistic posture re-education exhibited improvement in unstructured thoracic scoliosis. Guyot et al. (50) evaluated cervical proprioception in patients with idiopathic scoliosis. It was discovered that some individuals with AIS exhibit aberrant neck proprioception, which may portend unfavorable outcomes, including headaches, imbalance, and exacerbation of spinal deformities. However, not all studies concur that proprioceptive intervention has a beneficial effect on AIS. The study conducted by Müller-Gliemann et al. (51) did not observe any influence of proprioceptive insoles on body homeostasis or motion control. This may be due to the following reasons: (1) The etiology of AIS may stem not only from abnormalities in the proprioceptive system but also from other aspects of AIS neuromuscular control. Therefore, the likelihood of improving non-proprioceptive scoliosis through orthotic insoles is relatively low; (2) The form of proprioceptive abnormalities varies among different AIS patients. It is possible that these abnormalities represent secondary, erroneous proprioceptive patterns that develop after the onset of AIS. Thus, correction of ankle-foot proprioceptive input via orthotic insoles may not improve function in this subset of AIS patients.

## Discussion and conclusion

An anomalous proprioceptive system may precipitate the onset of AIS and may even exacerbate the condition as it progresses. The reacquisition of proprioception is thought to be an efficacious intervention for the recovery of AIS. In comparison to non-surgical treatment modalities, such as the use of a brace, passive manipulation and stretching, proprioceptive training is distinctive in that it is an active functional training approach. The majority of these programmes combine proprioceptive training with other forms of rehabilitation in order to intervene in AIS. This approach has been shown to result in improvements in both the Cobb angle and quality of life. The primary training methods for proprioception include active movement/balance training, passive movement training, somatosensory stimulation training, proprioceptive discrimination training, and combined/multisystem training. Training regimens lasting 6 weeks or longer typically yield relatively significant improvements in proprioception and/or motor function.

The current research on the proprioception of individuals with AIS primarily concentrates on the assessment of position perception, movement perception and overall limb proprioception. Some scholars posit that the intricate structure of the spine renders evaluation of the paraspinal muscles unfeasible. The somatosensory outcome measures included in the systematic review studies can be categorized into clinical and subclinical tests (14). The study employed the repositioning test (measuring active repositioning error) and the movement detection test (measuring the threshold for detecting passive movement) as clinical somatosensory tests. Body regions assessed included the neck, fingers, elbows, knees, and lower limbs. Additionally, the study measured latency and conduction velocity of somatosensory evoked potentials in the median and tibial nerves as subclinical proprioception tests.

However, the absence of local proprioception assessment constraints the investigation of whether AIS is characterised by spinal proprioceptive dysfunction. The emergence of certain emerging technologies or methods—such as advanced motion analysis or wearable sensor systems—may be able to compensate for this gap. Furthermore, the question of whether there is a causal relationship between sensory dysfunction and scoliosis requires further investigation.
